# A New Milk Combine

**Published:** 1920-08-21

**Authors:** 


### A New Milk Combine.

### WILL IT BENEFIT THE PUBLIC?

At a time when the cost of bread is going up and the cost of coal threatens to go up still further, the scheme which is being put on foot for making a huge milk combine of producers and retailers will be regarded with anxious concern by every section of the community. A very strong and extremely plausible case is put forward in behalf of such an organisation. It is to encourage the farmer who produces the milk; it will organise transportation on a co-operative basis, its improved efficiency will cut into heavy costs, and the interests of the middleman and the producer will be identical. This may be perfectly true. But what about the interests of the consumer? The point which the consumer will want to know is how far the increased efficiency and the diminished cost will benefit him, and how far the two advantages will be swallowed up in extra profits which will go to the shareholders of a great monopoly. This is a point of view which is entitled to obtain a hearing and a satisfactory answer. Milk is one of the vital necessities of life. Not only is every hospital in the country largely dependent for its ability to serve the sick on a pure and reasonably cheap milk supply, but milk is the touchstone of infantile well-being. The price of milk is already dangerously high. Let it go a little higher and it will seriously jeopardise the health of those elements in the nation which are most in need of strengthening.

We understand that the promoters claim to have safeguarded the public by means of a price-fixing committee, the chairman of which is to be an independent gentleman nominated by the Government. In our opinion, such a provision is far below the minimum of safety. The public, that is to say the consumers, should be represented directly on the committee by disinterested persons of their own selection and equal in number to the nominees of the milk-supplying organisations, and so give the independent chairman nominated by the Government the right to a casting-vote. That is the least the public can insist upon in face of a monopoly which, unchecked, might gravely embarrass many large institutions and those classes of society least able to protect themselves. All unrestricted monopolies are bad, but this one would be as bad as any we know of.

